# Supplementary material for: The relationship between gender discrimination and wellbeing in middle-aged and older women
Source: PLoS One. 2024 Mar 20;19(3):e0299381. doi: 10.1371/journal.pone.0299381 (PMC10954130; doi:10.1371/journal.pone.0299381)
Supplement: S1 Table — (DOCX) [file pone.0299381.s001.docx]

| **Supplementary Table 1:** Cross-sectional and prospective associations between perceived discrimination and health and wellbeing outcomes in the sample without complete body mass index data | | | | | | | | | | | | | |  |
| --- | --- | --- | --- | --- | --- | --- | --- | --- | --- | --- | --- | --- | --- | --- |
|  | |  |  |  | | **Wave 5** | | | **Wave 8** | | | | | |
|  | |  |  | **n** | **No perceived discrimination** | | **n** | **Perceived discrimination** |  | **n** | **No perceived discrimination** | **n** | **Perceived discrimination** | |
| Depression | | | |  |  | |  |  |  |  |  |  |  | |
|  | Mean score (SE) | | | 3671 | 1.63 (0.03) | | 370 | 1.92 (0.10) |  | 2577 | 1.52 (0.03) | 280 | 1.55 (0.10) | |
|  | Coeff. [95%CI] | | |  | Ref | |  | 0.29 [0.08; 0.50]** |  |  | Ref |  | 0.03 [-0.17; 0.24] | |
| Loneliness | | | |  |  | |  |  |  |  |  |  |  | |
|  | Mean score (SE) | | | 3660 | 1.43 (0.01) | | 367 | 1.56 (0.03) |  | 2353 | 1.37 (0.01) | 257 | 1.44 (0.03) | |
|  | Coeff. [95%CI] | | |  | Ref | |  | 0.13 [0.07; 0.18]*** |  |  | Ref |  | 0.07 [0.02; 0.12]** | |
| Quality of life | | | |  |  | |  |  |  |  |  |  |  | |
|  | Mean score (SE) | | | 3533 | 41.19 (0.14) | | 360 | 38.59 (0.43) |  | 2152 | 42.06 (0.13) | 247 | 41.23 (0.38) | |
|  | Coeff. [95%CI] | | |  | Ref | |  | -2.60 [-1.72; -3.48]*** |  |  | Ref |  | -0.82 [-1.61; -0.04]* | |
| Life satisfaction | | | |  |  | |  |  |  |  |  |  |  | |
|  | Mean score (SE) | | | 3568 | 20.49 (0.10) | | 365 | 19.34 (0.32) |  | 2237 | 20.86 (0.10) | 255 | 19.91 (0.30) | |
|  | Coeff. [95%CI] | | |  | Ref | |  | -1.15 [-1.81; -0.49]*** |  |  | Ref |  | -0.95 [-0.33; -1.57]** | |
| All analyses are adjusted for age, wealth, ethnicity, marital status, smoking and physical activity. Prospective analyses are additionally adjusted for baseline scores/status.  Coeff = unstandardized B coefficient, CI = confidence interval  **p*<0.05, ***p*<0.01, ****p*<0.001  Possible scores on the depression measure range from 0-8, on the loneliness measure range from 1-3, on the quality of life scale range from 0-57, and on the life satisfaction scale range from 0-30. | | | | | | | | | | | | | |  |
