# Supplementary material for: The relationship between gender discrimination and wellbeing in middle-aged and older women
Source: PLoS One. 2024 Mar 20;19(3):e0299381. doi: 10.1371/journal.pone.0299381 (PMC10954130; doi:10.1371/journal.pone.0299381)
Supplement: S2 Table — (DOCX) [file pone.0299381.s002.docx]

| **Supplementary Table 2:** Characteristics of complete cases and those lost to follow-up at wave 5 of the English Longitudinal Study of Ageing (2010/11). | | | | |
| --- | --- | --- | --- | --- |
|  |  | **Lost to follow-up**  **(*n* = 789)** | **Complete cases**  **(*n* = 2292)** | ***p*** |
| Age (years) | | 70.89 (10.06) | 66.15 (7.78) | < 0.001 |
| Ethnicity (% white) | | 788 (98.6%) | 2248 (98.1%) | = 0.336 |
| Marital status (% married) | | 417 (52.9%) | 1420 (62.0%) | < 0.001 |
| Wealth quintile (£) | |  |  | < 0.001 |
| 1 | | 171 (21.7%) | 341 (14.9%) |  |
| 2 | | 186 (23.6%) | 448 (19.5%) |  |
| 3 | | 154 (19.5%) | 461 (20.1%) |  |
| 4 | | 147 (18.6%) | 492 (21.5%) |  |
| 5 | | 131 (16.6%) | 550 (24.0%) |  |
| Body Mass Index (kg/m^2^) | | 27.95 (5.36) | 28.34 (5.64) | = 0.083 |
| Smoking (% yes) | | 112 (14.2%) | 236 (10.3%) | = 0.003 |
| Physical activity (% sedentary) | | 229 (29.0%) | 276 (12.0%) | < 0.001 |
| Data are presented as means (SD) and n (%)  Complete cases are defined as those who were present at wave 5 and provided data on at least one measure at wave 8. | | | |  |
