# Supplementary material for: The relationship between gender discrimination and wellbeing in middle-aged and older women
Source: PLoS One. 2024 Mar 20;19(3):e0299381. doi: 10.1371/journal.pone.0299381 (PMC10954130; doi:10.1371/journal.pone.0299381)
Supplement: S3 Table — (DOCX) [file pone.0299381.s003.docx]

| **Supplementary Table 3:** Cross-sectional and prospective associations between perceived discrimination and health and wellbeing outcomes in those who provided follow-up data | | | | | | | | | | | | | |
| --- | --- | --- | --- | --- | --- | --- | --- | --- | --- | --- | --- | --- | --- |
|  | |  |  |  | | **Wave 5** | | | **Wave 8** | | | | |
|  | |  |  | **n** | **No perceived discrimination** | | **n** | **Perceived discrimination** |  | **n** | **No perceived discrimination** | **n** | **Perceived discrimination** |
| Depression | | | |  |  | |  |  |  |  |  |  |  |
|  | Mean score (SE) | | | 2046 | 1.40 (0.04) | | 217 | 1.83 (0.12) |  | 2046 | 1.48 (0.04) | 217 | 1.56 (0.11) |
|  | Coeff. [95%CI] | | |  | Ref | |  | 0.43 [0.17; 0.68]*** |  |  | Ref |  | 0.08 [-0.16; 0.31] |
| Loneliness | | | |  |  | |  |  |  |  |  |  |  |
|  | Mean score (SE) | | | 1875 | 1.38 (0.01) | | 202 | 1.52 (0.03) |  | 1875 | 1.36 (0.01) | 202 | 1.44 (0.03) |
|  | Coeff. [95%CI] | | |  | Ref | |  | 0.14 [0.07; 0.21]*** |  |  | Ref |  | 0.08 [0.02; 0.14]* |
| Quality of life | | | |  |  | |  |  |  |  |  |  |  |
|  | Mean score (SE) | | | 1722 | 42.60 (0.19) | | 194 | 39.87 (0.56) |  | 1722 | 42.33 (0.14) | 194 | 41.36 (0.43) |
|  | Coeff. [95%CI] | | |  | Ref | |  | -2.74 [-1.59; -3.89]*** |  |  | Ref |  | -0.98 [-0.09; -1.86]* |
| Life satisfaction | | | |  |  | |  |  |  |  |  |  |  |
|  | Mean score (SE) | | | 1774 | 21.11 (0.14) | | 199 | 20.04 (0.41) |  | 1774 | 21.01 (0.11) | 199 | 19.97 (0.34) |
|  | Coeff. [95%CI] | | |  | Ref | |  | -1.07 [-1.91; -0.22]* |  |  | Ref |  | -1.04 [-0.34; -1.74]** |
| All analyses are adjusted for age, wealth, ethnicity, marital status, body mass index, smoking and physical activity. Prospective analyses are additionally adjusted for baseline scores/status.  Coeff = unstandardized B coefficient, CI = confidence interval  Possible scores on the depression measure range from 0-8, on the loneliness measure range from 1-3, on the quality of life scale range from 0-57, and on the life satisfaction scale range from 0-30. | | | | | | | | | | | | | |
