# Supplementary material for: The relationship between gender discrimination and wellbeing in middle-aged and older women
Source: PLoS One. 2024 Mar 20;19(3):e0299381. doi: 10.1371/journal.pone.0299381 (PMC10954130; doi:10.1371/journal.pone.0299381)
Supplement: S4 Table — (DOCX) [file pone.0299381.s004.docx]

| **Supplementary Table 4:** Cross-sectional and prospective associations between perceived discrimination and health and wellbeing outcomes (imputed) | | | | | | | | | | | | | |
| --- | --- | --- | --- | --- | --- | --- | --- | --- | --- | --- | --- | --- | --- |
|  | |  |  |  | | **Wave 5** | | | **Wave 8** | | | | |
|  | |  |  | **n** | **No perceived discrimination** | | **n** | **Perceived discrimination** |  | **n** | **No perceived discrimination** | **n** | **Perceived discrimination** |
| Depression | | | |  |  | |  |  |  |  |  |  |  |
|  | Mean score (SE) | | | 2765 | 1.55 (0.04) | | 281 | 1.89 (0.11) |  | 2765 | 1.61 (0.04) | 281 | 1.70 (0.12) |
|  | Coeff. [95%CI] | | |  | Ref | |  | 0.34 [0.11; 0.57]** |  |  | Ref |  | 0.10 [-0.15; 0.33] |
| Loneliness | | | |  |  | |  |  |  |  |  |  |  |
|  | Mean score (SE) | | | 2775 | 1.41 (0.01) | | 280 | 1.55 (0.03) |  | 2775 | 1.39 (0.01) | 280 | 1.47 (0.01) |
|  | Coeff. [95%CI] | | |  | Ref | |  | 0.14 [0.08; 0.20]*** |  |  | Ref |  | 0.07 [0.01; 0.13]** |
| Quality of life | | | |  |  | |  |  |  |  |  |  |  |
|  | Mean score (SE) | | | 2678 | 41.53 (0.15) | | 273 | 39.03 (0.48) |  | 2678 | 41.17 (0.14) | 273 | 40.17 (0.43) |
|  | Coeff. [95%CI] | | |  | Ref | |  | -2.50 [-1.51; -3.49]*** |  |  | Ref |  | -1.00 [-0.12; -1.88]* |
| Life satisfaction | | | |  |  | |  |  |  |  |  |  |  |
|  | Mean score (SE) | | | 2691 | 20.65 (0.12) | | 278 | 19.58 (0.36) |  | 2691 | 20.56 (0.11) | 278 | 19.55 (0.32) |
|  | Coeff. [95%CI] | | |  | Ref | |  | -1.07 [-1.81; -0.33]** |  |  | Ref |  | -1.00 [-0.35; -1.67]** |
| All analyses are adjusted for age, wealth, ethnicity, marital status, body mass index, smoking and physical activity. Prospective analyses are additionally adjusted for baseline scores/status.  Coeff = unstandardized B coefficient, CI = confidence interval  **p*<0.05, ***p*<0.01, ****p*<0.001  Possible scores on the depression measure range from 0-8, on the loneliness measure range from 1-3, on the quality of life scale range from 0-57, and on the life satisfaction scale range from 0-30. | | | | | | | | | | | | | |
