# Supplementary material for: The relationship between gender discrimination and wellbeing in middle-aged and older women
Source: PLoS One. 2024 Mar 20;19(3):e0299381. doi: 10.1371/journal.pone.0299381 (PMC10954130; doi:10.1371/journal.pone.0299381)
Supplement: S5 Table — (DOCX) [file pone.0299381.s005.docx]

|  | **Supplementary Table 5:**  Sensitivity analysis: Perceived discrimination measure excluding each discriminatory behaviour in turn | | | | | | |
| --- | --- | --- | --- | --- | --- | --- | --- |
| **Cross-sectional analyses (wave 5)** | | | **Model 1** | **Model 2** | **Model 3** | **Model 4** | **Model 5** |
| Depression | | Coeff. [95%CI] | 0.35 [0.14; 0.56]*** | 0.35 [0.12; 0.58]** | 0.34 [0.10; 0.58]** | 0.35 [0.12; 0.58]** | 0.34 [0.11; 0.57]** |
| Loneliness | | Coeff. [95%CI] | 0.15 [0.10; 0.21]*** | 0.15 [0.09; 0.21]*** | 0.14 [0.08; 0.21]*** | 0.14 [0.08; 0.21]*** | 0.14 [0.08; 0.20]*** |
| Quality of life | | Coeff. [95%CI] | -2.69 [-3.61; -1.76]*** | -2.63 [-3.64; -1.62]*** | -2.77 [-3.79; -1.75]*** | -2.53 [-3.53; -1.53]*** | -2.50 [-3.49; -1.50]*** |
| Life satisfaction | | Coeff. [95%CI] | -1.00 [-1.69; -0.31]** | -1.10 [-1.86; -0.35]** | -1.21 [-1.98; -0.45]** | -1.12 [-1.86; -0.37]** | -1.07 [-1.81; -0.33]** |
| **Prospective analyses (wave 8)** | | | **Model 1** | **Model 2** | **Model 3** | **Model 4** | **Model 5** |
| Depression | | Coeff. [95%CI] | 0.05 [-0.17; 0.27] | 0.09 [-0.15; 0.33] | 0.14 [-0.10; 0.38] | 0.09 [-0.15; 0.32] | 0.08 [-0.15; 0.31] |
| Loneliness | | Coeff. [95%CI] | 0.11 [0.05; 0.16]*** | 0.08 [0.02; 0.14]** | 0.08 [0.02; 0.14]* | 0.07 [0.01; 0.13]* | 0.08 [0.02; 0.14]* |
| Quality of life | | Coeff. [95%CI] | -0.75 [-1.58; 0.09] | -0.95 [-1.85; -0.06]* | -0.91 [-1.82; -0.01]* | -0.98 [-1.86; -0.09]* | -0.98 [-1.86; -0.09]* |
| Life satisfaction | | Coeff. [95%CI] | -1.15 [-1.82; -0.49]*** | -1.00 [-1.71; -0.30]** | -0.94 [-1.66; -0.23]** | -1.04 [-1.74; -0.34]** | -1.04 [-1.74; -0.34]** |
| All analyses are adjusted for age, wealth, ethnicity, marital status, body mass index, smoking and physical activity. Prospective analyses are additionally adjusted for baseline scores/status.  Model 1 excludes “*you are treated with less respect or courtesy*” from the measure of perceived age discrimination; Model 2 excludes “*you receive poorer service than other people in restaurants and stores*”; Model 3 excludes “*people act as if they think you are not clever*”; Model 4 excludes “*you are threatened or harassed*”; and Model 5 excludes “*you receive poorer service or treatment than other people from doctors or hospitals*”.  Coeff = unstandardized B coefficient, CI = confidence interval  **p*<0.05, ***p*<0.01, ****p*<0.001  Possible scores on the depression measure range from 0-8 on the loneliness measure range from 1-3, on the quality of life scale range from 0-57, and on the life satisfaction scale range from 0-30. | | | | | | | |
